# Applying Pulsed Electric Fields to Whole Carrots Enhances the Bioaccessibility of Carotenoid and Phenolic Compounds in Derived Products

**DOI:** 10.3390/foods10061321

**Published:** 2021-06-08

**Authors:** Gloria López-Gámez, Pedro Elez-Martínez, Olga Martín-Belloso, Robert Soliva-Fortuny

**Affiliations:** Department of Food Technology, University of Lleida, Agrotecnio Center, Av. Alcalde Rovira Roure, 191, 25198 Lleida, Spain; gloria.lopez@udl.cat (G.L.-G.); pedro.elez@udl.cat (P.E.-M.); olga.martin@udl.cat (O.M.-B.)

**Keywords:** carotenoids, phenolic compounds, puree, juice, bioaccessibility, pulsed electric fields, carrot, microstructure, quality attributes

## Abstract

We propose the application of pulsed electric fields (PEF) to carrots to obtain derived products with increased phenolic and carotenoid bioaccessibility. For this purpose, juices, purees, and oil-added purees were obtained from whole PEF-treated carrots (five pulses of 3.5 kV cm^−1^; 0.61 kJ kg^−1^). In order to obtain shelf-stable products, the effect of a thermal treatment (70 °C for 10 min) was also studied. Carrot juices exhibited the highest carotenoid (43.4 mg/100 g fresh weight) and phenolic (322 mg kg^−1^ dry weight) contents. However, caffeic and coumaric acid derivatives were highly sensitive to PEF. The phenolic bioaccessibility reached 100% in purees obtained from the PEF-treated carrots, whereas the further thermally treated oil-added purees exhibited the greatest carotenoid bioaccessibility (7.8%). The increase in carotenoid bioaccessibility could be related to their better release and solubilization into micelles. The results suggest that food matrix aspects apart from particle size (e.g., pectin characteristics) are involved in phenolic bioaccessibility.

## 1. Introduction

Today’s life pace leads consumers to increasingly demand healthier minimally processed products that are easy to prepare and consume. Carrots are one of the most consumed vegetables worldwide, and are, thus, a significant source of antioxidants, including carotenoids and phenolic compounds. Clinical studies have demonstrated that α-carotene and β-carotene, the most abundant carotenoids in carrots, can prevent suffering atherosclerosis, cancer, or macular degeneration [1]. Likewise, chlorogenic acid, the main phenolic compound found in carrots, possesses anti-diabetic and cardioprotective properties [2]. Therefore, due to their health-promoting properties, carrots are a potential commodity for developing functional derived products and meeting consumer demands.

Both carotenoids and phenolic compounds are usually enclosed by cell walls and organelle structures that hinder their release during digestion. Bioaccessibility refers to the percentage of a compound released from the food matrix and absorbed during digestion [3], which is more important than the actual content in a food matrix. The chemical structure, concentration, matrix structure, and processing are the most important factors that determine bioactive compound bioaccessibility [4]. Therefore, mechanical and thermal processes could disrupt the natural matrix, thus, modifying their further bioaccessibility [4,5]. 

A decrease in the particle size and depolymerization of pectin has been shown to improve β-carotene bioaccessibility in carrot purees [6,7,8]. Furthermore, carotenoid micellarization is conditioned by different factors, such as oil addition [9] and the application of thermal treatments [6]. Nonetheless, some studies have reported that cell wall fragments formed after thermal treatments may entrap carotenoids and compromise their bioaccessibility [10]. The bioaccessibility of phenolic compounds has been reported to increase in thermally treated grape and orange juices, whereas it was shown to decrease in pomelo (80 °C for 30 min) and fruit juice-based beverages (90 °C for 1 min) [11,12,13]. Information about the effect of the presence of oils and fats on phenolic bioaccessibility is limited since they are hydrophilic compounds that do not require micellarization prior to intestinal uptake. However, some literature works reported a positive effect when whole milk was added to juices [11,12,13].

Pulsed electric fields (PEF) is a non-thermal processing technology that delivers short pulses (ms or μs) of electric energy to a food product that is located between two electrodes. Electropermeabilization causes reversible or irreversible structural changes in the matrix depending on the applied intensity. Low and moderate intensities (0.1–5 kV cm^−1^, 0.5–20 kJ kg^−1^) have been reported to trigger a stress defense response in plant tissues, leading to the accumulation of bioactive compounds (e.g., carotenoids and phenolic compounds) in fruit and vegetables [14,15,16]. 

On the other hand, severe changes in structure may facilitate the extraction of bioactive compounds and, consequently, their bioaccessibility [17,18]. Results regarding the application of PEF to whole apples [19], tomatoes, and carrots to enhance the bioaccessibility of carotenoids and phenolic compounds are promising, suggesting that derivates, such as juices or purees, with higher and more bioaccessible antioxidant compounds, could be obtained from these commodities [20,21].

Therefore, the main aim of this study was to investigate the feasibility of applying PEF to whole carrots as a pre-treatment to enhance the bioaccessible fractions of carotenoids and phenolic compounds in different shelf-stabled derived products (juices, purees, and oil-added purees). Additionally, the influence of applying PEF and further processing strategies on the quality attributes, microstructure, and bioactive contents was investigated.

## 2. Materials and Methods

### 2.1. Chemicals and Reagents

HPLC grade methanol, acetone, and methyl tert-butyl ether were acquired from Fisher Scientific Scharlau Chemie (Loughborough, UK). Sodium chloride was purchased from POCH S.A. (Sowińskiego, Poland). Ultrapure water was obtained with a Milli-Q system (Millipore Ibérica, Madrid, Spain). Ammonium carbonate, acetonitrile, hexane, ethanol (HPLC grade), magnesium chloride hexahydrate, acetic acid, and ammonium acetate were acquired from Scharlab (Sentmenat, Spain). Butyl hydroxytoluene (BHT) was purchased from Scharlau Chemie S.A. (Barcelona, Spain). Calcium chloride dihydrate was obtained from Merck (Darmstadt, Germany). Sodium hydrogen carbonate and potassium dihydrogen phosphate were acquired from VWR (Llinars del Vallès, Spain). Potassium chloride was obtained from Panreac (Castellar del Vallès, Spain). Digestive enzymes (porcine pepsin, porcine bile extract, porcine pancreatin, and porcine lipase) were acquired from Sigma-Aldrich (Darmstadt, Germany). 

Caffeic acid, ferulic acid, and p-coumaric acid commercial patterns were obtained from Sigma-Aldrich (St. Louis, MO, USA). B-carotene standard was acquired from Carote-Nature (Ostermundigen, Switzerland), and α-carotene was acquired from Supelco-Merck (Darmstadt, Germany).

### 2.2. Carrot Samples

Carrots (*Daucus carota* cv. Nantes) (17 ± 2 cm and 106 ± 7 g) were purchased in a local supermarket (Lleida, Spain) and stored at 4 °C within a week until processing. Carrots were washed with tap water, and the excess was removed with a paper cloth before PEF application.

### 2.3. Pulsed Electric Fields (PEF) Treatments

PEF treatments were conducted in a batch PEF system (Physics International, San Leandro, CA, USA) equipped with a TG-70 gas control unit and a PT55 pulse generator (Pacific Atlantic Electronics Inc., El Cerrito, CA, USA). The system delivers exponential pulses of 4 μs from a capacitor of 0.1 μF at a frequency of 0.1 Hz. The treatment chamber consists of a parallelepiped methacrylate container with two parallel stainless-steel electrodes (20 × 5 cm) separated by a gap of 5 cm. Whole carrots (0.1 kg) were immersed in an aqueous solution (conductivity of 10 μS cm^−1^) and placed in parallel to the electrodes. Then, carrots were subjected to five pulses of 3.5 kV cm^−1^ (0.61 kJ kg^−1^) and were stored at 4 °C for 24 h. The treatment conditions were selected based on previous results in which phenolic and carotenoid bioaccessibilities were enhanced in whole carrots [20].

The specific energy input was calculated based on Wiktor et al. [22], and the medium temperature was measured after treatment application to ensure that it remained constant after PEF application.

### 2.4. Preparation of Carrot Derived Products

Untreated and PEF-treated carrots were washed with tap water, and the excess was removed with a paper cloth before discarding their top and bottom ends. Two types of puree were obtained: one batch from untreated (U) and the other from PEF-treated carrots (PEF). Purees were prepared by mixing approximately 500 g of 1-cm thick carrot slices with water (1:1) (*w*/*w*) in a food processor (Taurus Mycook) operated with the crushing function at full power in two 10-s intervals. 

To prepare oil-added carrot purees, extra virgin olive oil (Borges Branded Foods, S.L.U., Tàrrega, Lleida, Spain) was added (5% *w*/*w*), and the homogenates were stirred for 15 min at 8000 rpm with an Ultra-Turrax IKA equipped using a 3-blade stirring rod. The olive oil included 0.86 mg/100 g fresh weight (FW) of α-carotene, 2.59 mg/100 g FW of β-carotene, 0.18 mg kg^−1^ dry weight (DW) of coumaric acid, 0.02 mg kg^−1^ DW of caffeic acid, and 0.02 mg kg^−1^ DW of ferulic and isoferulic acids.

Two types of carrot juices were obtained from approximately 500 g of 1-cm thick carrot slices using a cold blender (Imetec Succovivo SJ1000 coupled to a filter of 0.4 mm). One batch was obtained from untreated carrots and another from PEF-treated carrots (0.61 kJ kg^−1^). The resulting purees and juices were divided into two fractions, that were thermally treated (U/T or PEF/T) or remained unheated (U or PEF) as a reference for the former treatments.

Thermal treatment was applied in order to inactivate pectin methylesterase and peroxidase activities [23,24,25], thus, obtaining stable products. Carrot purees or juices (200 g) were packed in re-sealable polyethylene bags (20 × 15 cm) and heated in a water bath for 10 min at 70 °C. The product temperature was monitored during treatment to assure that the purees/juices did not exceed 70 °C. Thereafter, the purees were cooled under a constant flow of cold water for 3 min. Aliquots (20 mL) of the non-digested fractions were stored at −40 °C until the extraction and analysis of carotenoids were performed. Then, the samples were freeze-dried in order to extract the phenolic content. Additional aliquots (20 mL) were subjected to an in vitro digestion to determine their carotenoid and phenolic contents in digesta.

### 2.5. Evaluation of Quality Attributes 

The color was evaluated by measuring the CIEL* a* and b* parameters with a colorimeter (Minolta CR-400, Konica Minolta Sensing, INC., Osaka, Japan). The total color difference (ΔE) was also calculated using Equation (1).

ΔE = [[(L* − L*_0_)^2^ + (a* − a*_0_)^2^ + (b* − b*_0_)^2^]]^0.5^ (1)

where L*_0_, a*_0_, and b*_0_ refer to untreated carrot products and L*, a*, and b* correspond to the data collected after treatments.

The pH was assessed in products using a pH meter (Crison Instruments S.A., Alella, Barcelona, Spain). The total soluble solids (TSS) was measured using a refractometer (Atago Company Ltd., Tokyo, Japan) and expressed as % of the total soluble solids. 

### 2.6. Particle Size Distribution 

A Mastersizer 3000™ (Malvern Instruments Ltd., Worcestershire, UK) was used to measure the particle size distribution of juices and purees. The results were expressed in terms of the volume and surface diameter, D [4, 3] and D [3, 2], respectively. The refractive index of water was 1.33, and particle calculation was set for irregular particles.

### 2.7. Microstructure

The microstructure was investigated using a light microscope (BX41, Olympus, Göttingen, Germany) equipped with UIS2 optical system. We mounted 10 μL drops on glass slides without staining and microscopically observed them. A general inspection of the samples was made, and representative photos were taken with the 10× lens. All images were processed using the instrument software Olympus CellSense (Barcelona, Spain). 

### 2.8. In Vitro Digestion 

The in vitro digestion procedure was performed according to the standardized COST Infogest protocol [26], in which electrolyte and enzymatic solutions to simulate the phases of human digestion are described. Digestions were performed in darkness, in the absence of oxygen (bottles were flushed with nitrogen gas) in an orbital incubator (Ovan, Badalona, Spain) at 37 °C and 120 rpm. Electrolyte concentrations and enzyme activities were prepared following the indications provided by Minekus et al. [26], and blank samples consisting of water instead of carrot products, were made in identical conditions.

The oral phase was omitted due to the very short residence times of purees and juices in the oral cavity [26]. Then, the gastric phase started by adding 20 mL of simulated gastric fluid (pH 3 and 37 °C) and pepsin to 20 g of juice/puree. This mixture was incubated at 37 °C for 2 h in agitation. The duodenal phase was initiated by inserting a cellulose-membrane dialysis bag (molecular weight cut-off 12,000 Da, Sigma-Aldrich, St. Louis, MO, USA), which contained simulated intestinal fluid (pH 7 and 37 °C). This dialysis bag simulates the intestinal epithelium, and it harbors phenolic compounds released from the matrix (bioaccessible fraction) [19,26]. 

After 30 min of incubation, the pH was adjusted to 7, and a solution containing simulated intestinal fluid (pH 7 and 37 °C), bile extract, pancreatin (and lipase in case of purees containing oil) was added and the mixture was further incubated for 2 h. At the end of digestion, the dialysis bags were rinsed with distilled water until clean, and its contents were collected. The remaining digesta, which contained carotenoid compounds, was centrifuged at 5000× *g* for 15 min at 4 °C [27,28,29], and the supernatant was also collected. Digested fractions were freeze-dried and stored at −40 °C until analysis. 

### 2.9. Carotenoids Determination

#### 2.9.1. Carotenoids Extraction

Carotenoids were extracted following the method described by Sadler et al. [30], with slight modifications. An extraction solution (50 mL) composed of hexane:acetone:ethanol (50:25:25) and 1 g·L^−1^ BHT was added to carrot puree (2 g) or juice (1 g) and was stirred for 20 min. Then, 15 mL of NaCl (10% (*w*/*v*)) solution was added, and the samples were stirred for 10 additional minutes. The samples were left to stand for ≥3 minutes, and the upper organic phase was microfiltered across a nylon filter (0.45 μm, ø 13 mm, Labbox Labware S.L., Barcelona, Spain) and analyzed using High-Performance Liquid Chromatography with Diode Array Detection (HPLC-DAD).

Recovery of the carotenes from the micellar digested fraction was performed by adding 5 mL of the extraction solution to 0.2 g of freeze-dried digesta. After that, the samples were vortexed for 20 s, and 1 mL of NaCl solution (10% (*w*/*v*)) was added. The samples were vortexed for another 20 s and centrifuged at 4000× *g* for 5 min [29]. An aliquot of the upper organic phase was microfiltered across a nylon filter (0.45 μm, ø 13 mm, Labbox Labware S.L., Barcelona, Spain) and analyzed by HPLC using the same method as for non-digested fractions.

All extractions were performed in duplicate, and the samples were protected from light throughout extraction and analysis to avoid carotenoid degradation and isomerization. 

#### 2.9.2. Identification and Quantification of Carotenoids by HPLC-DAD 

The carotenoids were quantified by HPLC-DAD, following a procedure validated by Cortés et al. [31]. The HPLC system was equipped with a 600 Controller, a 486 Absorbance Detector, a thermostatic column compartment, and a 717 Plus Auto Sampler with a cooling system (Waters, Milford, MA, USA). An aliquot of 20 µL of the extracted samples was injected and the carotenoids were separated using a reverse-phase C18 Spherisorb ODS2 (5 µm) stainless steel column (4.6 × 250 mm). 

The mobile phase consisted of: (A) methanol/ammonium acetate 0.1 M, (B) milli-Q water, (C) methyl tert-butyl ether, and (D) methanol. The flow rate was fixed at 1 mL min^−1^, and the total run time was 60 min. The column was set at 30 °C, while sample amber vials on the autosampler were preserved at 4 °C. Identification was carried out by UV–vis spectral data and their retention times [31]. Carotenoids were quantified by using calibration curves and integrating peak areas. The results are expressed on a fresh weight basis.

### 2.10. Phenolic Compounds Determination

#### 2.10.1. Phenolic Compounds Extraction 

Phenolic compounds were extracted from freeze-dried non-digested or digested carrot puree/juice (0.2 g). For non-digested juices and digested fractions of purees and juices, 1 mL of methanol (80:20 *v*/*v*) was added, whereas 1.5 mL was needed for the non-digested purees. The samples were vortexed for 1 min and then centrifuged (16,209× *g*, 15 min, 4 °C). The clear supernatant was microfiltered using polyvinylidene difluoride (PVDF) filters (0.2 μm) (Scharlab, Barcelona, Spain) prior to injection into the chromatographic system. 

#### 2.10.2. Identification and Quantification of Phenolic Compounds by Ultra-Performance™ Liquid Chromatography (UPLC-MS/MS) 

Phenolic compounds and their generated metabolites were determined in methanolic extracts obtained from freeze-dried non-digested or digested fractions. AcQuity Ultra-Performance™ liquid chromatography (UPLC) coupled to a triple quadrupole detector (TQD) mass spectrometer (Waters, Milford, MA, USA) was used. The analytical column was an AcQuity BEH C18 column (100 × 2.1 mm i.d., 1.7 μm), equipped with a VanGuard™ Pre-Column AcQuity BEH C18 (2.1 × 5 mm, 1.7 μm). During the analysis, the column was kept at 30 °C, and the flow rate was 0.3 mL min^−1^. 

Mobile phases were acetic acid (0.2%) and acetonitrile (Table 1). Tandem MS analyses were carried out on a triple quadrupole detector (TQD) mass spectrometer (Waters, Milford, MA, USA) equipped with a Z-spray electrospray interface (ESI). Ionization was achieved using the electrospray interface operating in the negative mode [M−H]^−^, and the data were acquired through selected reaction monitoring (SRM). The dwell time established for each transition was 30 ms. Data acquisition was carried out with the MassLynx 4.1 software (Milford, MA, USA).

### 2.11. Bioaccessibility Calculation 

The bioaccessibility of individual compounds was determined using Equation (2), and results are expressed as a percentage.

Bioaccessibility (%) = CC_digested_/CC_non-digested_ × 100 (2)

where CC_digested_ corresponds to the overall concentration of each compound in the absorbable fraction and CC_non-digested_ is the concentration in non-digested samples.

The carotenoid bioaccessibility was calculated referring to the concentration found in the digested micellar fraction, whereas the phenolic compound bioaccessibility was calculated in reference to their concentration in the dialyzed digested fraction. 

### 2.12. Statistical Analysis 

Statistical analyses were carried out using the SigmaPlot 11.0 software (Systat Software Inc., Chicago, IL, USA). Three different replicates were submitted to each assayed treatment condition, and each analysis was conducted thrice, excepting the extraction and determination of phenolics and carotenoids, which were conducted twice. The results are reported as the mean ± standard deviation and were subjected to an analysis of variance (ANOVA) followed by the Tukey post hoc test to establish statistical differences among treatments. A two-way ANOVA was carried out for establishing differences between derived products. In the case where the results showed no homogeneity in their variance, they were subjected to ANOVA on ranks by the Kruskal–Wallis test. The statistical significance level was set up at *p* < 0.05.

## 3. Results

### 3.1. Quality Attributes

The quality attributes from differently treated carrot juices and purees are shown in Table 2. The TSS and pH were not generally influenced either by PEF treatment or by further processing conditions, although the color was differently affected. PEF application to carrot matrix did not cause significant changes in the L* or a* values of any carrot-derived product, although b* was lower in juices. Hence, the ΔE value of PEF purees was 1.6, whereas that of PEF juices reached 3.8. In the latter case, color changes would be visually apparent, as ΔE > 2 have been reported to be noticeable by consumers [32]. Similar results were reported by Aguiló-Aguayo et al. [33] and Xiang et al. [34] in PEF-treated carrot purees and juices, respectively.

Oil addition and thermal treatment also affected the color of different products. On the one hand, oil addition increased the values of the CIEL*a*b* coordinates compared to purees without added oil. These differences would be detectable by consumers, given that ΔE values were between 3.3 and 4.7. On the other hand, the temperature did not affect the L* nor b* in any derived product. However, thermally treated juices (U/T and PEF/T) exhibited higher a* values than those untreated, and the ΔE values were higher than 2. Moreover, U/T and PEF/T oil-added purees had lower a* compared with untreated ones. 

Color differences could be associated with the disruption of cells and the breakage of the chromoplast carotenoid–protein complexes, leading to the release of carotenoids [33,35]. Likewise, non-enzymatic Maillard reactions could also be responsible for color changes after thermal treatments, since carrot juice had greater sugar contents, and high temperatures could favor this reaction [36]. In addition, Mutsokoti et al. [37] demonstrated that carotenoid transfer to oil was enhanced by thermal treatments, although carotenoid degradation was also more pronounced in the presence of oil [38], which could explain the decrease in a* values.

### 3.2. Particle Size Distribution

The mean particle size of differently treated carrot-based products is displayed in Table 2. Purees were constituted by 3–5% of particles smaller than 100 μm, which may suggest that they were mainly formed by cell fragments or single cells since carrot cell size has been reported to average ca. 125 μm [7]. On the other hand, juices had between 13–17% of particles below 100 μm, which is likely due to the removal of cell clusters during juicing. Hence, juices mainly contained small fragments of disrupted tissue and cells.

PEF treatment before further processing did not cause significant changes in the mean particle size of any derived product. Untreated and PEF juices were characterized by D [3, 2] of 66 ± 17 μm and 74.3 ± 0.9 μm, respectively. Otherwise, untreated and PEF purees showed D [3, 2] = 207 ± 48 μm and 183 ± 34 μm, respectively. Some authors reported that PEF treatment may facilitate juice-pressing due to structural rearrangements. Thus, derived products with smaller particle sizes than those untreated could be obtained [39]. However, the results from this work suggest that PEF treatment was not intense enough to cause a lower resistance to mechanical load and promote changes in particle size. Similarities in particle size distribution between PEF and untreated products could indicate that they will have similar stability during storage. 

Thermal treatments (U/T and PEF/T) caused changes in the area-based mean diameter of juices and purees without oil, whereas those with oil added were not affected. Purees showed higher D [3, 2] values (472 ± 20 and 431 ± 53 μm) compared to those of untreated purees (207 ± 48 μm). This increase in the size of small particles after thermal treatments has been reported in thermally treated tomato suspensions [40], which was attributed to the swelling of cells or the formation of aggregates from cellular components [10]. 

However, it could also mean that the largest cell clusters were disintegrated in smaller cell aggregates, which increased D [3, 2]. On the other hand, juices (U/T and PEF/T) had between 29–34% of particles below 100 μm and D [3, 2] in the range of 32–46 μm. These results indicate that thermal treatments increased the number of small particles, reducing the mean particle size (D [3, 2]). This was also observed in pasteurized orange juices [41,42]. Thermal treatments between 65 and 95 °C have been reported to cause disruptions between chromoplast membrane structures and/or carotenoid protein interactions, which could cause a decrease in the particle size of juices [10,43].

On the other hand, the particle sizes (D [4, 3] and D [3, 2]) of oil-added purees were significantly lower than that of purees without oil. Oil-added purees had between 29–37% of particles below 100 μm. These values indicate that purees would be mainly formed by cell fragments or single cells together with oil droplets (20 μm) [7]. Any treatment affected the oil-added purees particle size. These results were likely highly influenced by the presence of oil droplets, which likely has a major influence on the particle size distribution and masked changes in the particle size of purees.

### 3.3. Microstructure

**Figure 1:**
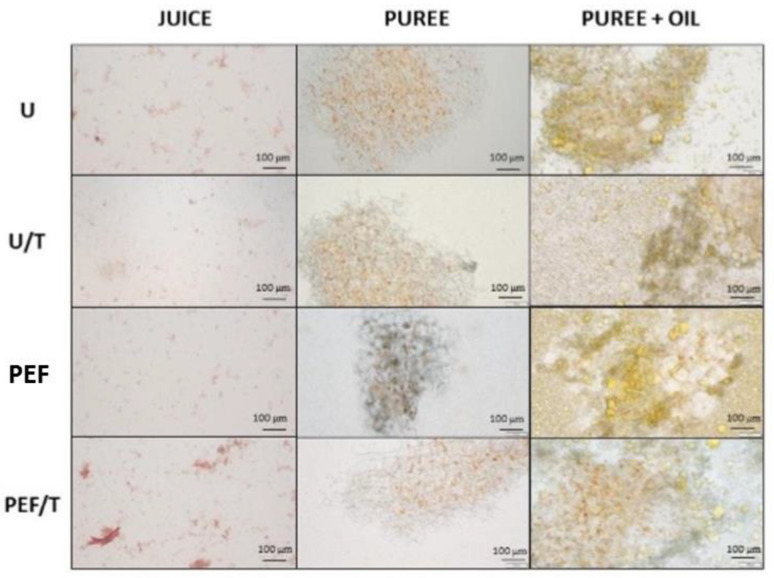
Representative light micrographs of juices and purees obtained from untreated carrots (U), obtained from PEF-treated (0.61 kJ kg^−1^) carrots (PEF) and thermally treated (10 min at 70 °C) (U/T and PEF/T).

The microstructure of juices, purees, and oil-added purees is shown in Figure 1. Both purees showed clusters of whole cells with carotenoids inside. On the other hand, whole cells were rarely detected in juices (Figure 1) given that cells and chromoplasts are likely comminuted during processing. Juices are mainly composed of cloud particles of different densities formed by chromoplast fragments and carotenoids, which was verified through isopycnic gradient centrifugation by Marx et al. [44]. 

The application of PEF to whole carrots did not cause significant changes in the microstructure of the obtained juices. However, PEF purees showed some starch grains inside cells that were absent in untreated purees. Gómez Galindo et al. [45] demonstrated that PEF can trigger changes in the hexose pool due to stress induction. Specifically, it could have affected the AGPase activity, which is involved in starch biosynthesis, or starch-degrading enzymes [46]. To the best of our knowledge, microstructural studies of derived products obtained from PEF-treated matrices are limited. However, some authors have observed irregular cell walls, such as folds and loss of smoothness in PEF-treated whole tomatoes and carrots [18,47].

Thermal treatment of the derived products was shown to lead to the presence of thin cell walls in purees, probably as a consequence of depolymerization and pectin degradation [48]. Additionally, temperature likely favors interactions between dissolved particles [49], which would explain the increases in particle size, D [3, 2] (Table 2). On the other hand, thermally treated juices had a greater content of small particles than those untreated or PEF. Temperatures between 65–95 °C are able to break carotenoid-chromoplasts complexes [43], which could explain the decrease in particle size (Table 2) and increased carotenoid release (Figure 1).

The oil-added purees showed lipid droplets surrounding carrot clusters (Figure 1). Oil was especially internalized in thermally treated and PEF purees, given that the disruption of cell membranes may facilitate the efflux and influx of intra- and extracellular content. These results have been previously reported in excipient emulsions of olive oil and tomato by Li et al. [50], who attributed this lipid entry to capillary forces generated by pores in tissues [51].

### 3.4. Carotenoid Content

**Figure 2:**
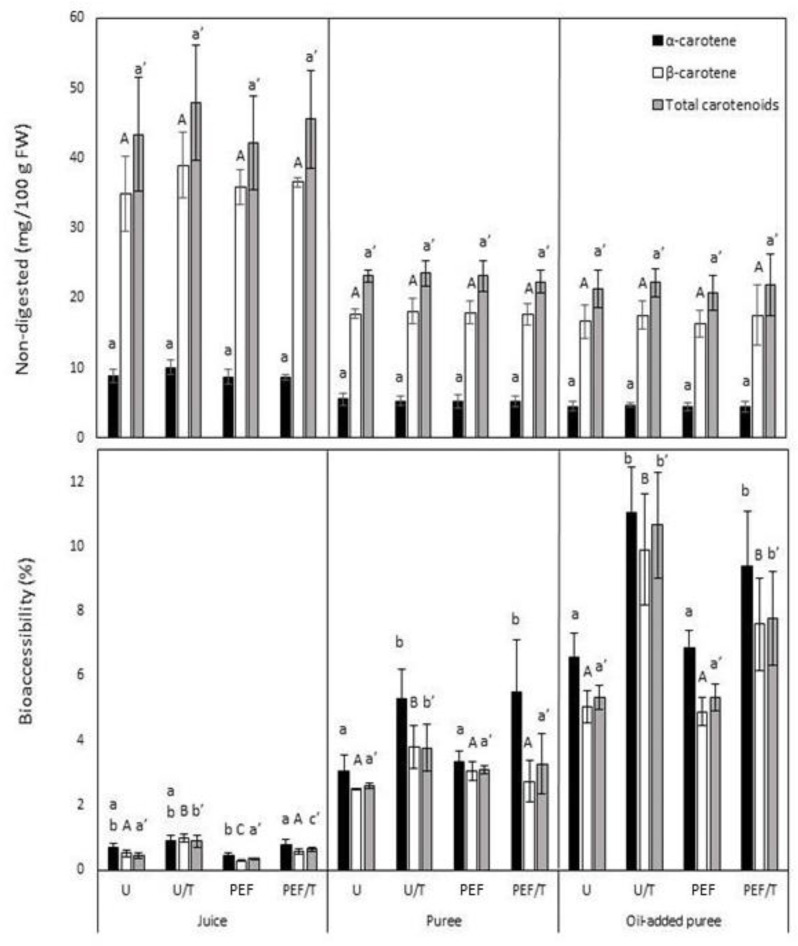
The carotenoid content and bioaccessibility of untreated purees and juices (U), those obtained from PEF-treated (0.61 kJ kg^−1^) carrots (PEF), and those thermally treated (10 min at 70 °C) (U/T and PEF/T). Values are means ± standard deviation. Different letters within the same product and compound indicate significant (*p* < 0.05) differences among treatments (α-carotene: lowercase letters; β-carotene: uppercase letters; and total carotenoids: lowercase′).

The carotenoid contents from differently treated products are shown in Figure 2. PEF application to whole carrots did not affect the content nor carotenoid profile, although further processing caused a significant effect in carotenoid concentration. Hence, different carotenoid contents were obtained depending on the type of derived product; however, neither thermal treatment nor oil addition caused significant changes. Carrot juices had higher carotenoid contents (43.4 ± 7–48 ± 8 mg/100 g FW) compared with both types of puree (20.7 ± 2.5–23.6 ± 1.8 mg/100 g FW). However, these differences were mainly related to dilution during the puree preparation. The obtained results are in accordance with Hedrén et al. [8], given that mechanical processing was more determinant to the release of carotenoids than thermal treatment or oil addition.

The main carotenoids present in carrot-derived products were α-carotene and β-carotene. These results are in agreement with those previously reported [52,53], although some authors have also detected lutein presence in carrots. Panozzo et al. [54] hypothesized that crystalloid chromoplasts were more easily disrupted by mechanical processing compared with globular chromoplasts, in which lutein is usually dissolved. In addition, lutein is more susceptible to degradation due to the presence of oxygen in its chemical structure [55]. Hence, it could be degraded during mechanical processing or be entrapped in chromoplasts.

The carotenoid contents of derived products obtained from PEF-treated whole matrices have been scarcely studied. However, increases in the carotenoid content of purees and juices obtained from PEF-treated tomatoes have been reported [18,21]. Such increases were attributed to two main causes: (1) their accumulation in the whole product resulting from the induction of a stress defense response and (2) their better extractability due to electropermeabilization. On the other hand, Rybak et al. [39] observed a decreased content in juices obtained from PEF-treated peppers (3 kJ kg^−1^), which was attributed to promoted oxidation or isomerization reactions. Therefore, our results suggest that selected PEF conditions did not induce carotenoid biosynthesis during 24 h of storage nor improve their extractability in carrot-based products.

Thermal treatment did not enhance the carotenoid extractability nor induce their degradation in any derived product. Similar results were obtained in thermally treated oil-added carrot purees [6]. However, decreases in carrot juices [56] and increases in carrot purees [57] have been reported. Differences between our results and those previously mentioned are likely due to variations in the processing parameters (time or temperature) or derived product preparation procedures. These processing conditions likely also caused differences in particle size, structural properties, or enzyme activities, which are closely related to carotenoid extractability or degradation.

### 3.5. Carotenoid Bioaccessibility

The carotenoid bioaccessibility was affected by PEF treatment and further processing. Mechanical processing was the main factor that influenced bioaccessibility, followed by oil addition and thermal treatment (Figure 2). The highest total bioaccessibility was obtained in oil-added purees (5.3%), whereas purees (2.6%) and juices (0.4%) had lower bioaccessibility. 

The application of PEF to carrots before obtaining derived products did not affect the total bioaccessibility. Generally, thermal treatments were those that led to further enhancement of the total bioaccessibility: oil-added purees (10.7%) > purees (3.8%) > juices (0.9%). Regarding the bioaccessibility of individual carotenoids, α-carotene and β-carotene similarly increased in any of the studied products as a consequence of the application of thermal treatments, whereas PEF only caused a decrease in β-carotene bioaccessibility when it was applied before juicing (Figure 2).

Regarding the carotenoid bioaccessibility in derived products obtained from PEF-treated carrots, they contrast with those reported by González-Casado et al. [21]. The authors presented increases in the total carotenoids and β-carotene bioaccessibility of purees obtained from PEF-treated tomatoes, which were attributed to electropermeabilization and better carotenoids release. The initial content of such tomato purees was considerably higher than in the untreated samples, which could make a difference regarding our study since the content in non-digested products was similar regardless of the applied treatment. 

In addition, an observed decrease in the β-carotene bioaccessibility from juices was also reported by Bot et al. [17] in PEF-treated tomato chromoplast fractions. This was attributed to induced modifications in carotenoid-protein complexes, which limit their bioaccessibility. Finally, some authors have also suggested that released carotenoids can be broken down during digestion into non-detected metabolites (e.g., oxidation products) [58].

Carotenoids are generally located inside chromoplasts or bound to the membrane [59]. Hence, chromoplasts are probably comminuted during juicing or blending, which makes carotenoids more available to be absorbed when the particle size decreases as a consequence of processing (Table 2). Apart from a particle size decrease, carotenoids bioaccessibility also depends on their chemical structure, interactions with other macromolecules, micellarization, content, and the characteristics of pectin [60]. The increase in bioaccessibility after thermal treatments has been related to carotenoid isomerization [44], given that cis isomers are better assimilated than trans [61]. On the other hand, thermal processing can degrade pectin and may improve carotenoid bioaccessibility [48,62], given that a high pectin content would entrap carotenoids or act as a barrier for lipase [63], hindering their micellarization. 

The solubilization of carotenoids into micelles is another critical step in carotenoid absorption. The low amount of lipids in purees and juices could hinder their micellarization, given that carotenoids are not water-soluble. Hence, the presence of lipids facilitates carotenoid transference to micelles [64]. The obtained results indicate that the application of thermal treatment to oil-added purees caused the highest bioaccessibility. Previous studies have shown that thermal-treated carrot cell clusters (95–110 °C) improved carotenoid transfer to oil [65].

### 3.6. Phenolic Content

The phenolic content was affected by both PEF treatment and further processing (Table 3). Carrot juices showed the highest phenolic content (322 ± 56 mg kg^−1^ DW), whereas oil-added purees (81 ± 36 mg kg^−1^ DW) and purees (62 ± 23 mg kg^−1^ DW) exhibited similar contents. PEF treatment applied to whole carrots generally did not affect the phenolic content of both types of puree, although it decreased the content in juices (38.5%). Despite this reduction, the phenolic content in juices remained higher than in purees. On the other hand, the total phenolic content in purees without added oil was doubled when a thermal treatment was applied, whereas the contents in juices and oil-added purees were not significantly affected. 

The main phenolic compounds found in carrot-derived products were hydroxycinnamic acids, namely 5-caffeoylquinic acid, coumaroylquinic acid or 5-feruloylquinic acid (Table 3). These results are in accordance with previous studies performed in whole carrots [20,66] or juices [67,68]. It has been reported that a low dietary fiber content in juices is beneficial for releasing phenolic compounds [69]. Furthermore, the extractability of phenolic compounds may be enhanced in juices due to their lower particle size (Table 2). Likewise, differences in phenolic composition between purees and juices could also be related to mechanical processing. The procedure applied to obtain juices may have enhanced the extraction of compounds tightly linked to cell walls, whereas carrot cells were not totally disrupted in purees. 

PEF treatment caused different effects depending on the type of carrot-derived product and phenolic chemical structure. Hence, the application of PEF to whole carrots did not affect most of the compounds from both purees obtained from such carrots, although the juices had lower contents of coumaric, ferulic, and caffeic acid derivatives (e.g., 5-caffeoylquinic (68.5%)) (Table 3). The selected PEF treatment was based on previous results obtained by López-Gámez et al. [70] in which the total phenolic content of whole carrots was enhanced. Nevertheless, such an increase was not observed in derived products obtained from PEF-treated whole carrots. This may suggest that mechanical processing could affect the phenolic stability or favor their interactions with cell wall debris, which would hinder their extractability.

The results indicate that individual compounds were also differently affected by thermal treatment, depending on their structure and carrot-derived product. Hence, caffeic acid derivatives were strongly enhanced in purees (e.g., 5-caffeoylquinic acid (231%)), although caffeic acid decreased in those with oil added (Table 3). Juices showed increases in some caffeic acids derivates (e.g., 3-caffeoylquinic acid (400%)); however, a lower content was observed in some ferulic acid derivates (e.g., 5-feruloylquinic acid). Increases in phenolic content have been previously reported in thermally treated fruit juices (e.g., caffeoyl glucoside or caffeic acid) [11,12,13]. This was attributed to their better release due to cell wall disruptions, which is in agreement with the lower particle sizes observed in the juices (Table 2). The higher content in caffeic acid derivatives could result from the partial inactivation of enzymes responsible for phenolic degradation (e.g., polyphenol oxidase, PPO) [13].

### 3.7. Phenolic Bioaccessibility

The phenolic compound bioaccessibility was affected by PEF treatment application to whole carrots and further processing. The bioaccessibility differed depending on the evaluated carrot-derived product. Carrot purees had the highest total bioaccessibility (52%) followed by oil-added purees (31%) and juices (16.1%) (Table 4). PEF application to carrots before mechanical processing caused a large enhancement of the total bioaccessibility, hence, reaching 100% in purees, whereas that of juices and oil-added purees was not affected. On the other hand, thermal treatment did not significantly influence the total bioaccessibility in any carrot-based product (Table 4). 

Individual compounds were differently affected depending on their chemical structure and processing. PEF treatments increased the bioaccessibility of most ferulic (e.g., ferulic acid glucoside) and caffeic acid derivatives (e.g., 5-caffeoylquinic acid) in purees, although some decreases were also observed (e.g., coumaric acid). Oil-added purees were similarly affected to those without lipids; however, the bioaccessibility of certain compounds decreased (e.g., 5-caffeoylquinic acid or feruloylquinic acid derivate (2)). 

On the other hand, the individual phenol bioaccessibility in juices obtained from PEF-treated carrots was not affected, excepting that of isoferulic acid, which reached 100%. Limited information about the phenolic bioaccessibility in juices and purees obtained from PEF-treated matrices is available in the literature. Generally, phenolic compounds should be easily released from juices, due to their low content in dietary fiber [69]. However, the obtained results are controversial since the phenolic bioaccessibility in carrot juice was the lowest (Table 4). 

The juices had a lower particle size (D [4, 3] = 487 μm) compared with the purees (D [4, 3] = 596 μm), which favored phenolic release from the matrix. Phenolic compounds are likely more exposed to degradation or entrapment by other macromolecules during digestion in juices than in purees, hence, limiting their bioaccessibility [71]. PEF application to carrots strongly enhanced the bioaccessibility in purees without oil, whereas treatment was not effective for juices or oil-added purees. Despite no correlation being found between the particle size and bioaccessibility values, cell permeability changes could have caused a better release and dialysis of phenols in purees. In addition, variations caused by PEF in the initial phenolic content of carrots would directly affect the bioaccessibility in carrot-derived products.

## 4. Conclusions

The content and bioaccessibility of carotenoids and phenolic compounds were affected by both PEF application to whole carrots and further processing conditions. Carrot juices had the highest phenolic and carotenoid content. However, only the phenolic content in purees further increased after a thermal treatment, whereas it decreased in juices obtained from PEF-treated carrots. On the other hand, PEF pre-treatment of whole carrots stands as a potential method for enhancing the phenolic bioaccessibility in purees since most of them were completely dialyzed after treatment. 

Nevertheless, PEF was not helpful to improve the total phenolic bioaccessibility in purees with added oil and juices. Regarding carotenoids, PEF pre-treatment did not substantially enhance their total bioaccessibility, whereas thermal treatment and oil addition were more effective to improve the carotenoid bioaccessibility in carrot-based purees. Therefore, these results demonstrate that applying PEF as a pre-treatment is a feasible strategy for developing products with an enhanced nutritive value. Further studies focused on the matrix structure and composition (e.g., pectin characteristics) are necessary to understand the mechanisms governing changes in the bioaccessibility of health-related compounds in the different studied products.

## Figures and Tables

**Table 1:** Chromatographic conditions for phenolic compounds identification by UPLC.

Time (min)	Acetic Acid (0.2% *v*/*v*) (%)	Acetonitrile (%)
0	95	5
5	90	10
10	87.6	12.4
18	72	28
21	15	85
23	0	100
25.5	0	100
27	95	5
30	95	5

**Table 2:** Quality attributes and particle size of untreated carrot purees and juices (U), those obtained from PEF-treated (0.61 kJ kg^−1^) carrots (PEF), and those thermally treated (10 min at 70 °C) (U/T and PEF/T).

Product	Treatment	L*	a*	b*	ΔE	pH	TSS (%)	D [4, 3] (μm)	D [3, 2] (μm)
Puree	U	41.3 ± 0.3 ^a,A^	11.0 ± 0.3 ^a,A^	24.4 ± 0.2 ^a,A^	-	6.4 ± 0.1 ^a,b,c,A^	3.6 ± 0.3 ^a,A^	596 ± 12 ^a,A^	207 ± 48 ^a,A^
	PEF	40.5 ± 0.1 ^a,A^	10.3 ± 0.1 ^a,A^	23.1 ± 0.2 ^a,A^	1.6 ± 0.2 ^a,A^	6.5 ± 0.1 ^b,A^	3.4 ± 0.2 ^a,A^	589 ± 16 ^a,A^	183 ± 34 ^a,A^
	U/T	42.0 ± 0.2 ^a,A^	10.4 ± 0.5 ^a,A^	24.3 ± 0.5 ^a,A^	1.3 ± 0.1 ^a,A^	6.1 ± 0.1 ^d,A^	3.7 ± 0.0 ^a,A^	608 ± 11 ^a,A^	472 ± 20 ^b,A^
	PEF/T	41.0 ± 0.4 ^a,A^	9.9 ± 0.5 ^a,A^	23.1 ± 0.8 ^a,A^	1.8 ± 0.7 ^a,A^	6.3 ± 0.0 ^c,A^	3.5 ± 0.3 ^a,A^	601 ± 12 ^a,A^	431 ± 53 ^b,A^
Oil-added puree	U	56.0 ± 1.7 ^a′,B^	15.2 ± 0.7 ^a′,B^	44.8 ± 1.9 ^a′,B^	-	6.3 ± 0.0 ^a′,A^	4.0 ± 0.6 ^a′,A^	449 ± 41 ^a′,B^	15 ± 3 ^a′,B^
	PEF	55.7 ± 0.3 ^a′,B^	14.5 ± 0.5 ^a′,b′,B^	45.6 ± 3.9 ^a′,B^	3.3 ± 2.4 ^a′A^	6.4 ± 0.1 ^a′,A^	3.8 ± 0.4 ^a′,A^	422 ± 15 ^a′,B^	13.8 ± 2.0 ^a′,B^
	U/T	55.3 ± 0.7 ^a′,B^	13.8 ± 0.4 ^b′,B^	45.6 ± 3.9 ^a′,B^	3.6 ± 1.9 ^a′,B^	6.0 ± 0.0 ^b′,A^	4.2 ± 0.4 ^a′,A^	460 ± 30 ^a′,B^	20 ± 4 ^a′,B^
	PEF/T	55.2 ± 0.7 ^a′,B^	12.4 ± 0.9 ^d′,B^	47 ± 3 ^a′,B^	4.7 ± 2.1 ^a′,B^	6.1 ± 0.0 ^c′,B^	3.9 ± 0.1 ^a′,A^	408 ± 39 ^a′,B^	16 ± 4 ^a′,B^
Juice	U	43.0 ± 0.5 ^a^^″^	15.8 ± 0.7 ^a^^″,b″^	29.2 ± 0.7 ^a^^″^	-	6.2 ± 0.1 ^a^^″^	8.2 ± 0.5 ^a^^″^	487 ± 43 ^a^^″^	66 ± 17 ^a^^″,b″^
	PEF	41.3 ± 0.3 ^a^^″^	13.4 ± 0.7 ^a^^″,b^^″^	26.9 ± 0.7 ^b^^″^	3.8 ± 1.9 ^a^^″^	6.2 ± 0.1 ^a^^″^	8.2 ± 06 ^a^^″^	499 ± 4 ^a^^″^	74.3 ± 0.9 ^a^^″^
	U/T	40.7 ± 2.3 ^a^^″^	18.6 ± 1.6 ^c^^″^	28.1 ± 1.3 ^a^^″,b^^″^	4.8 ± 1.7 ^a^^″^	6.1 ± 0.0 ^a^^″^	7.7 ± 0.4 ^a^^″^	359 ± 48 ^b^^″^	32 ± 2 ^b^^″^
	PEF/T	41.7 ± 0.3 ^a^^″^	17.9 ± 0.6 ^c^^″^	27.7 ± 0.7 ^a^^″,b^^″^	2.86 ± 0.2 ^a^^″^	6.3 ± 0.2 ^a^^″^	7.5 ± 0.2 ^a^^″^	408 ± 61 ^b^^″^	46 ± 3 ^b^^″^

Values are means ± standard deviation. Different letters in the same column within the same product indicate significant (*p* < 0.05) differences among treatments (puree: lowercase letters, puree with oil: lowercase letters′, and juice: lowercase″). Different uppercase letters in the same row indicate significant differences between both purees. L*: lightness, a*: redness, b*: yellowness, ΔE: total color difference, TSS: total soluble solids, D [4, 3]: mean volume diameter, and D [3, 2]: mean surface diameter.

**Table 3:** Phenolic content in untreated purees and juices (U), those obtained from PEF-treated (0.61 kJ kg^−1^) carrots (PEF), and those thermally treated (10 min at 70 °C) (U/T and PEF/T).

Phenolic Compounds	Puree				Oil-Added Puree				Juice			
	U	PEF	U/T	PEF/T	U	PEF	U/T	PEF/T	U	PEF	U/T	PEF/T
Coumaric acid	5.05 ± 0.21 ^a^	7.17 ± 1.82 ^b^	2.91 ± 0.50 ^c^	1.03 ± 0.24 ^d^	4.33 ± 0.58 ^A^	2.84 ± 0.30 ^B,C^	1.67 ± 0.04 ^C^	2.16 ± 0.71 ^C^	0.70 ± 0.07 ^a′^	0.79 ± 0.04 ^a′^	0.71 ± 0.13 ^a′^	0.75 ± 0.05 ^a′^
Coumaroylquinic acid	17 ± 8 ^a^	8 ± 3 ^a,b^	9.0 ± 2.5 ^a,b^	5.9 ± 1.23 ^b^	3.22 ± 0.60 ^A^	3.6 ± 0.74 ^A^	6.2 ± 2.3 ^A^	10 ± 4 ^A^	15 ± 7 ^a′^	6.01 ± 0.37 ^b′^	19 ± 3 ^a′^	6.41 ± 0.48 ^b′^
Coumaric acid and its derivates	22 ± 8 ^a^	15 ± 5 ^a,b^	12 ± 3 ^a,b^	6.9 ± 1.5 ^b^	7.6 ± 0.8 ^A^	6.4 ± 1.0 ^A^	7.9 ± 2.2 ^A^	12 ± 4 ^A^	15 ± 7 ^a′^	6.80 ± 0.36 ^b′^	19 ± 4 ^a′^	7.2 ± 0.5 ^b′^
Caffeic acid	1.49 ± 0.92 ^a^	0.83 ± 0.13 ^a^	0.92 ± 0.30 ^a^	0.42 ± 0.07 ^b^	1.72 ± 0.64 ^A^	0.61 ± 0.12 ^B^	0.51 ± 0.24 ^B^	0.39 ± 0.07 ^B^	1.3 ± 0.2 ^a′^	0.90 ± 0.11 ^a′^	2.47 ± 0.34 ^b′^	1.13 ± 0.10 ^a′^
Caffeic acid arab/xiloside	nd ^1,a^	nd ^1,a^	nd ^a^	nd ^a^	nd ^1,A^	nd ^1,A^	nd ^1,A^	nd ^1,A^	0.12 ± 0.03 ^a′^	0.06 ± 0.01 ^a′^	0.29 ± 0.06 ^b′^	0.14 ± 0.02 ^a′^
Caffeoylshikimic acid	nd ^1,a^	nd ^1,a^	nd ^1,a^	nd ^1,a^	nd ^1,A^	nd ^1,A^	nd ^1,A^	nd ^1,A^	0.06 ± 0.02 ^a′^	0.02 ± 0.01 ^b′^	0.05 ± 0.01 ^a′^	0.02 ± 0.003 ^b′^
3-caffeoylquinic acid	nd ^1,a^	nd ^1,a^	0.18 ± 0.08 ^b^	0.23 ± 0.02 ^b^	nd ^1,A^	nd ^1,A^	nd ^1,A^	0.08 ± 0.01 ^A^	0.21 ± 0.02 ^a′^	0.14 ± 0.02 ^a′^	0.97 ± 0.11 ^b′^	0.57 ± 0.09 ^c′^
5-caffeoylquinic acid	32 ± 17 ^a^	15.1 ± 1.0 ^a^	106 ± 23 ^b^	85 ± 13 ^b^	67 ± 35 ^A^	65 ± 20 ^A^	79 ± 6 ^A^	59 ± 19 ^A^	264 ± 47 ^a′^	83 ± 7 ^b′^	264 ± 32 ^a′^	92 ± 7 ^c′^
4-caffeoylquinic acid	nd ^1,a^	nd ^1,a^	1.73 ± 0.66 ^b^	1.62 ± 0.09 ^b^	0.17 ± 0.10 ^A^	0.30 ± 0.12 ^A^	0.8 ± 0.1 ^B^	0.65 ± 0.09 ^B^	0.76 ± 0.14 ^a′^	0.34 ± 0.07 ^a′^	7.66 ± 1.15 ^b′^	3.4 ± 0.3 ^c′^
Dicaffeoylferuoylquinic acid	nd ^1,a^	nd ^1,a^	0.25 ± 0.10 ^b^	0.21 ± 0.03 ^b^	nd ^1,A^	nd ^1,A^	nd ^1,A^	nd ^1,A^	0.31 ± 0.06 ^a′^	0.06 ± 0.01 ^b′^	0.55 ± 0.07 ^c′^	0.07 ± 0.02 ^b′^
Caffeoylferuoylquinic acid	0.35 ± 0.10 ^a^	0.38 ± 0.07 ^a^	0.29 ± 0.11 ^a^	0.35 ± 0.08 ^a^	0.24 ± 0.05 ^A^	0.14 ± 0.02 ^A^	0.23 ± 0.09 ^A^	0.18 ± 0.05 ^A^	0.48 ± 0.02 ^a′^	0.56 ± 0.05 ^b′^	0.43 ± 0.06 ^a′^	0.62 ± 0.06 ^b′^
Caffeic acid arabinoside glucoside	0.06 ± 0.02 ^a^	0.1 ± 0.02 ^a^	0.07 ± 0.01 ^a^	0.11 ± 0.02 ^a^	0.05 ± 0.002 ^A^	0.07 ± 0.02 ^A^	0.05 ± 0.02 ^A^	0.07 ± 0.01 ^A^	0.12 ± 0.06 ^a′^	0.18 ± 0.01 ^b′^	0.09 ± 0.02 ^a′^	0.22 ± 0.03 ^b′^
Caffeic acid Glu Acetyl glucoside	0.84 ± 0.46 ^a^	0.7 ± 0.19 ^a^	0.8 ± 0.5 ^a^	0.78 ± 0.07 ^a^	0.5 ± 0.04 ^A^	1.0 ± 0.35 ^A^	0.8 ± 0.14 ^A^	1.04 ± 0.13 ^A^	3.8 ± 0.2 ^a′^	4.32 ± 0.24 ^a′,b′^	3.34 ± 0.57 ^a′^	4.7 ± 0.4 ^b′^
Caffeic acid and its derivates	35 ± 17 ^a^	17.2 ± 1.3 ^a^	111 ± 23 ^b^	89 ± 13 ^b^	69 ± 35 ^A^	67 ± 20 ^A^	81 ± 5 ^A^	62 ± 19 ^A^	271 ± 48 ^a′^	90 ± 7 ^b′^	280 ± 34 ^a′^	103 ± 7 ^c′^
Ferulic acid	0.32 ± 0.07 ^a^	0.40 ± 0.15 ^a^	0.35 ± 0.14 ^a^	0.40 ± 0.02 ^a^	0.32 ± 0.03 ^A^	0.2 ± 0.02 ^A^	0.54 ± 0.18 ^A^	0.49 ± 0.05 ^A^	1.21 ± 0.12 ^a′^	1.69 ± 0.14 ^a′^	2.90 ± 0.55 ^b′^	2.64 ± 0.19 ^b′^
Isoferulic acid	0.08 ± 0.04 ^a^	0.10 ± 0.03 ^a^	0.07 ± 0.03 ^a^	0.08 ± 0.02 ^a^	0.12 ± 0.04 ^A^	0.06 ± 0.02 ^A^	0.09 ± 0.03 ^A^	0.10 ± 0.02 ^A^	0.20 ± 0.03 ^a′^	0.09 ± 0.02 ^b′^	0.18 ± 0.05 ^a′,b′^	0.11 ± 0.02 ^b′^
3-feruloylquinic acid	nd ^1,a^	nd ^1,a^	0.17 ± 0.03 ^b^	0.13 ± 0.02 ^b^	0.11 ± 0.06 ^A^	0.13 ± 0.02 ^A^	0.16 ± 0.03 ^A^	0.12 ± 0.03 ^A^	0.94 ± 0.12 ^a′^	0.45 ± 0.10 ^b′^	0.9 ± 0.12 ^a′^	0.67 ± 0.05 ^b′^
5-feruloylquinic acid	1.34 ± 0.41 ^a^	1.26 ± 0.05 ^a^	2.49 ± 0.46 ^a^	2.43 ± 0.19 ^a^	1.07 ± 0.16 ^A^	1.18 ± 0.1 ^A^	1.35 ± 0.27 ^A^	1.48 ± 0.47 ^A^	13.15 ± 1.92 ^a′^	8.90 ± 0.37 ^b′^	9.73 ± 1.20 ^b′^	9.2 ± 0.8 ^b′^
4-feruloylquinic acid	0.11 ± 0.01 ^a^	0.14 ± 0.01 ^a^	0.33 ± 0.06 ^a^	0.3 ± 0.04 ^a^	0.13 ± 0.0 ^A^	0.1 ± 0.09 ^A^	0.18 ± 0.04 ^A^	0.17 ± 0.02 ^A^	1.74 ± 0.25 ^a′^	1.25 ± 0.04 ^b′^	1.62 ± 0.28 ^a′^	1.56 ± 0.11 ^a′^
Ferulic acid glucoside	nd ^1,a^	nd ^1,a^	0.05 ± 0.03 ^a^	0.05 ± 0.004 ^a^	nd ^1,A^	nd ^1,A^	nd ^1,A^	0.49 ± 0.25 ^B^	0.32 ± 0.03 ^a′^	0.25 ± 0.02 ^a^′	0.29 ± 0.06 ^a′^	0.19 ± 0.01 ^a′^
Ferulic acid coumaroyl glucoside	2.02 ± 0.50 ^a^	1.46 ± 0.21 ^a^	1.72 ± 0.43 ^a^	1.41 ± 0.19 ^a^	1.52 ± 0.24 ^A^	1.44 ± 0.84 ^A^	2.15 ± 0.48 ^A^	1.32 ± 0.02 ^A^	3.72 ± 0.35 ^a′^	3.44 ± 0.21 ^a′^	2.48 ± 0.42 ^b′^	1.65 ± 0.08 ^c′^
Ferulic acid caffeoyl glucoside	0.09 ± 0.03 ^a^	0.07 ± 0.03 ^a^	0.18 ± 0.12 ^a^	0.14 ± 0.08 ^a^	0.06 ± 0.03 ^A^	nd ^1,A^	0.08 ± 0.03 ^A^	0.07 ± 0.03 ^A^	8.56 ± 0.61 ^a′^	4.31 ± 0.31 ^b′^	7.3 ± 1.0 ^a′,c′^	6.95 ± 0.53 ^c′^
Feruloylquinic acid derivative	0.85 ± 0.18 ^a^	0.8 ± 0.1 ^a^	0.68 ± 0.33 ^a^	0.83 ± 0.10 ^a^	0.43 ± 0.15 ^A^	0.4 ± 0.02 ^A^	0.43 ± 0.01 ^A^	0.42 ± 0.05 ^A^	2.92 ± 0.25 ^a′^	4.25 ± 0.10 ^b′^	2.52 ± 0.42 ^a′^	2.42 ± 0.23 ^a′^
Feruloylquinic acid derivative (2)	0.2 ± 0.08 ^a^	0.1 ± 0.02 ^a^	0.23 ± 0.10 ^a^	0.11 ± 0.01 ^a^	0.09 ± 0.03 ^A^	0.11 ± 0.01 ^A^	0.12 ± 0.01 ^A^	0.11 ± 0.02 ^A^	1.20 ± 0.11 ^a′^	1.34 ± 0.05 ^a′^	0.9 ± 0.12 ^b′^	1.34 ± 0.1 ^a′^
Ferulic acid and its derivatives	5.09 ± 0.12 ^a^	4.4 ± 0.3 ^a^	6.3 ± 1.3 ^a^	5.9 ± 0.3 ^a^	3.9 ± 0.4 ^A^	3.7 ± 0.7 ^A^	5.1 ± 0.8 ^A^	4.8 ± 0.8 ^A^	36 ± 4 ^a′^	26.8 ± 1.0 ^b′^	30 ± 4 ^b′^	27.8 ± 1.9 ^b′^
Total phenolic compounds	62 ± 23 ^a^	37 ± 5 ^a^	129 ± 25 ^b^	101 ± 14 ^b^	81 ± 36 ^A^	77 ± 20 ^A^	94 ± 6 ^A^	79 ± 24 ^A^	322 ± 56 ^a′^	124 ± 8 ^b′^	329 ± 40 ^a′^	138 ± 9 ^b′^

Values are means ± standard deviation. Different letters within the same product and compound indicate significant (*p* < 0.05) differences among treatments (puree: lowercase letters; puree with oil: uppercase letters; and juice: lowercase′). nd ^1^: not detected.

**Table 4:** Phenolic bioaccessibility of untreated purees and juices (U), those obtained from PEF-treated (0.61 kJ kg^−1^) carrots (PEF), and those thermally treated (10 min at 70 °C) (U/T and PEF/T).

Phenolic Compounds	Puree				Oil-Added Puree				Juice			
	U	PEF	U/T	PEF/T	U	PEF	U/T	PEF/T	U	PEF	U/T	PEF/T
Coumaric acid	91 ± 11 ^a^	56 ± 12 ^b^	53 ± 10 ^b^	100 ± 0 ^a^	63 ± 4 ^A^	99.4 ± 1.1 ^B^	79 ± 7 ^A^	77 ± 28 ^A^	100 ± 0 ^a′^	100 ± 0 ^a′^	47 ± 7 ^b′^	45.1 ± 1.1 ^b′^
Coumaroylquinic acid	80 ± 20 ^a^	100 ± 0 ^b^	100 ± 0 ^b^	100 ± 0 ^b^	84 ± 14 ^A^	97.4 ± 2.2 ^B^	42.7 ± 0.4 ^C^	26.3 ± 2.2 ^D^	42 ± 4 ^a′^	35.9 ± 2.1 ^a′^	25 ± 4 ^b′^	50 ± 4 ^c′^
Coumaric acid and its derivatives	84 ± 18 ^a^	96.2 ± 6.5 ^a^	100 ± 0 ^a^	100 ± 0 ^a^	74 ± 8 ^A^	100 ± 0 ^B^	48.8 ± 0.1 ^C^	31.2 ± 0.2 ^D^	56 ± 6 ^a′^	60 ± 3 ^a′^	25 ± 4 ^b′^	50 ± 4 ^a′^
Caffeic acid	0 ^a^	0 ^a^	52 ± 32 ^b^	24 ± 4 ^b^	0 ^A^	0 ^A^	43 ± 13 ^B^	24 ± 5 ^C^	0 ^a′^	0 ^a′^	47 ± 7 ^b′^	34 ± 5 ^b′^
Caffeic acid arab/xiloside	0* ^a^	0* ^a^	0 ^a^	0 ^a^	0 *^,A^	0 *^,A^	0 *^,A^	84.4 ± 18.6 ^B^	0 ^a′^	0 ^a′^	23 ± 7 ^b′^	55 ± 9 ^c′^
Caffeoylshikimic acid	0 ^a^	0 ^a^	0 ^a^	0 ^a^	0 ^A^	0 ^A^	0 ^A^	0 ^A^	0 ^a′^	0 ^a′^	0 ^a′^	0 ^a′^
3-caffeoylquinic acid	0 *^,a^	0 *^,A^	100 ± 0 ^b^	100 ± 0 ^b^	0 *^,A^	0 *^,A^	0 *^,A^	0 *^,A^	0 ^a′^	0 ^a′^	0 ^a′^	0 ^a′^
5-caffeoylquinic acid	15 ± 9 ^a^	56 ± 10 ^b^	22 ± 4 ^a^	12 ± 4 ^a^	16 ± 11 ^A^	8.4 ± 1.6 ^B^	29 ± 3 ^A^	28 ± 11 ^A^	0 ^a′^	0 ^a′^	26.1 ± 1.7 ^b′^	23 ± 5 ^b′^
4-caffeoylquinic acid	100 ± 0 ^a^	100 ± 0 ^a^	100 ± 0 ^a^	66 ± 6 ^b^	100 ± 0 ^A^	73 ± 35 ^B^	100 ± 0 ^A^	100 ± 0 ^A^	10.6 ± 1.8 ^a′^	19 ± 5 ^a′^	93 ± 6 ^b′^	84 ± 14 ^b′^
Dicaffeoylferuoylquinic acid	0 ^a^	0 ^a^	0 ^a^	0 ^a^	0 ^A^	0 ^A^	0 ^A^	0 ^A^	0 ^a′^	0 ^a′^	18 ± 5 ^b′^	0 ^a′^
Caffeoylferuoylquinic acid	39 ± 5 ^a^	69 ± 16 ^b^	46 ± 14 ^a^	66 ± 18 ^a,b^	48 ± 17 ^A^	48 ± 12 ^A^	36 ± 12 ^A^	25.9 ± 1.7 ^A^	67 ± 5 a′	47 ± 1.7 ^a′,b′^	43 ± 13 ^a′,b′^	28.7 ± 1.8 ^b′^
Caffeic acid arabinoside glucoside	73 ± 27 ^a^	55.2 ± 2.4 ^a^	62 ± 6 ^a^	48.0 ± 2.4 ^a^	84 ± 27 ^A^	42 ± 28 ^B^	100 ± 0 ^A^	71 ± 22 ^A^	74 ± 29 ^a′^	57 ± 3 ^a′^	86 ± 8 ^a′^	35.8 ± 1.9 ^a′^
Caffeic acid Glu acetyl glucoside	89 ± 19 ^a^	100 ± 0 ^a^	99.1 ± 1.5 ^a^	100 ± 0 ^a^	100 ± 0 ^A^	100 ± 0 ^A^	100 ± 0 ^A^	100 ± 0 ^A^	55 ± 3 ^a′^	45 ± 7 ^a′^	35 ± 9 ^a′^	31.9 ± 0.9 ^a′^
Caffeic acid and its derivatives	19 ± 9 ^a^	66 ± 9 ^b^	26 ± 5 ^a^	16 ± 4 ^a^	20 ± 13 ^A,B^	11.7 ± 2.3 ^B^	33 ± 3 ^A^	33 ± 12 ^A^	1.3 ± 0.2 ^a′^	3.4 ± 0.4 ^a′^	29.1 ± 1.9 ^b′^	27 ± 5 ^b′^
Ferulic acid	99 ± 0.7 ^a^	100 ± 0 ^a^	76 ± 24 ^b^	99.2 ± 1.3 ^a^	100 ± 0 ^A^	100 ± 0 ^A^	100 ± 0 ^A^	95.1 ± 8.5 ^A^	100 ± 0 ^a′^	100 ± 0 ^a′^	68 ± 15 ^b′^	45 ± 6 ^c′^
Isoferulic acid	100 ± 0 ^a^	100 ± 0 ^a^	98 ± 4 ^a^	100 ± 0 ^a^	100 ± 0 ^A^	100 ± 0 ^A^	100 ± 0 ^A^	100 ± 0 ^A^	70 ± 16 ^a′^	100 ± 0 ^b′^	91.3 ± 15 ^b′^	99.2 ± 1.3 ^b′^
3-feruloylquinic acid	100 ± 0 ^a^	100 ± 0 ^a^	100 ± 0 ^a^	100 ± 0 ^a^	93.8 ± 11 ^A,B^	81 ± 19 ^B^	100 ± 0 ^A^	100 ± 0 ^A^	79 ± 11 ^a′^	100 ± 0 ^a′^	98 ± 4 ^a′^	98.0 ± 2.0 ^a′^
5-feruloylquinic acid	81 ± 17 ^a^	99.6 ± 0.7 ^b^	95 ± 5 ^a,b^	96 ± 4 ^a,b^	100 ± 0 ^A^	91 ± 9 ^A^	100 ± 0 ^A^	100 ± 0 ^A^	40 ± 6 ^a′^	43 ± 3 ^a′^	56 ± 9 ^b′^	35 ± 5 ^a′^
4-feruloylquinic acid	100 ± 0 ^a^	100 ± 0 ^a^	100 ± 0 ^a^	100 ± 0 ^a^	100 ± 0 ^A^	100 ± 0 ^A^	100 ± 0 ^A^	99.5 ± 0.8 ^A^	86 ± 12 ^a′,b′^	83 ± 7 ^a′,b′^	94 ± 5 ^b′^	66 ± 6 ^c′^
Ferulic acid glucoside	56 ± 4 ^a^	99.1 ± 1.5 ^b^	100 ± 0 ^b^	91 ± 15 ^b^	0 ^A^	0 ^A^	60.5 ± 35 ^B^	8 ± 3 ^C^	34.7 ± 0.9 ^a′^	38 ± 6 ^a′^	36 ± 8 ^a′^	37 ± 7 ^a′^
Ferulic acid coumaroyl glucoside	71 ± 19 ^a^	100 ± 0 ^a^	76 ± 20 ^a^	94 ± 12 ^a^	81 ± 24 ^A^	57 ± 37 ^A^	78 ± 22 ^A^	82 ± 24 ^A^	44 ± 8 ^a′^	41.2 ± 1.7 ^a′^	99 ± 3 ^b′^	57.9 ± 1.9 ^a′^
Ferulic acid caffeoyl glucoside	38 ± 15 ^a^	65 ± 3 ^b^	100 ± 0 ^c^	92 ± 14 ^c^	71 ± 31 ^A^	92 ± 14 ^A^	61 ± 14 ^A^	78 ± 19 ^A^	36 ± 3 ^a′^	43.3 ± 0.9 ^a′^	37 ± 7 ^a′^	24 ± 7 ^a′^
Feruloylquinic acid derivative	46 ± 11 ^a^	89 ± 9 ^b^	89 ± 19 ^b^	93 ± 12 ^b^	73 ± 27 ^A^	78 ± 10 ^A^	68 ± 14 ^A^	78 ± 9 ^A^	46 ± 7 ^a′^	33 ± 3 ^a′^	41 ± 14 ^a′^	33.5 ± 0.3 ^a′^
Feruloylquinic acid derivative (2)	18 ± 7 ^a^	57 ± 12 ^b^	53 ± 24 ^b^	89 ± 19 ^c^	77 ± 21 ^A^	42 ± 17 ^B^	39 ± 17 ^B^	38 ± 10 ^B^	38.3 ± 2.4 ^a′^	33.6 ± 1.2 ^a′^	31 ± 5 ^a′^	19 ± 4 ^b′^
Ferulic acid and its derivatives	77 ± 4 ^a^	100 ± 0 ^b^	97 ± 5 ^b^	100 ± 0 ^b^	98 ± 3 ^A^	89 ± 12 ^A^	100 ± 0 ^A^	100 ± 0 ^A^	45 ± 4 ^a′^	50.0 ± 1.8 ^a′,b′^	60 ± 10 ^b′^	37 ± 5 ^a′^
Total phenolic compounds	52 ± 14 ^a^	100 ± 0 ^b^	49 ± 8 ^a^	48 ± 6 ^a^	31 ± 15 ^A^	24 ± 5 ^A^	40 ± 4 ^A^	40 ± 17 ^A^	16.1 ± 2.5 ^a′^	27.9 ± 2.0 ^a′^	34 ± 3 ^a′^	33 ± 2 ^a′^

Values are means ± standard deviation. Different letters within the same product and compound indicate significant (*p* < 0.05) differences among treatments (puree: lowercase letters; puree with oil: uppercase letters; and juice: lowercase′). The asterisk (*) indicates that the compound was detected in the dialyzed fraction, but it was not present in non-digested fractions.
